# The Cyclopædia of Practical Medicine

**Published:** 1834-01-01

**Authors:** 


*The Cyclopædia of Practical Medicine.* Edited by JOHN FORBES, M.D., ALEXANDER TWEEDIE, M.D., and JOHN CONOLLY, M.D. Parts XVIII. and XIX.—London, 1833. 8vo. pp. 256.*

THE first of the present parts begins with the conclusion of Dr. MONTGOMERY's article on *the Signs of Pregnancy and Delivery.* The author insists very strongly on the areola as a diagnostic mark, agreeing with the writers (Smellie and Hunter, for instance,) who regard it as the consequence of utero-gestation alone; and he gives a curious case in which this sign led him to detect a concealed pregnancy. Dr. M. says, "In the centre of this circle the nipple is observed partaking of the altered colour of the part, and appearing turgid and prominent; and the part of the areola more immediately around the base of the nipple has its surface rendered unequal by the prominence of the glandular follicles, which, varying in number from twelve to twenty, project from the sixteenth to the eighth of an inch; and, lastly, the integument covering the part is observed to be softer and more moist than that which surrounds it, and the breasts themselves are at the same time observed to be full and firm, at least more so than was natural to the person previously. Such we believe to be the essential characters of the true areola, the result of pregnancy, and that, when found possessing these marks, it ought to be looked on as the result of that condition alone, no other cause being capable of producing it."

The whole article is elaborate and instructive, but we can afford to give only one more short extract. The author is speaking of the sudden subsidence of an enlarged abdomen, which is often unjustly taken to be a proof of delivery:

"The writer very lately saw such an instance in the case of a woman separated from her husband, who became affected with what was considered ovarian dropsy, and which enlarged the abdomen to the size of six months' pregnancy, some of the other symptoms of which state were also present. After an attack of inflammation, during which it is to be presumed the parietes of the tumour formed

* We have likewise been favoured with copies of the article on the Signs of Pregnancy and Delivery, by Dr. Montgomery, and the one on Pseudo-morbid Appearances, by Dr. Todd.—EDITOR.

an adhesion with the upper part of the vagina, there took place suddenly a discharge of gelatinous fluid from that cavity, and the abdomen completely subsided in the course of a day, and the previously entertained suspicion appeared to be confirmed beyond a doubt; but on examination the woman had not about her one of the signs of delivery; yet, had not the case been at once investigated, loss of reputation at the least would have inevitably, though most undeservedly, followed." (P. 502.)

Dr. BEATTY has an article on *Rape*, in which very judicious observations in some passages are intermingled with others betraying a want of common sense, and written as a monk or a child might write. For instance:

"It is not impossible, nay, it has sometimes happened, that a woman who has freely consented to surrender her virtue will afterwards turn round on her paramour, and denounce him as her ravisher. This becomes a case of the greatest intricacy, from the fact of the principal feature (that of the venereal congress having taken place,) being true. It now passes out of the hands of the medical jurist, and becomes a question with the jury whether they believe the deposition of the woman as to consent or not. This, it must be confessed, is a most difficult question to solve, and it requires all the ingenuity of the bar to sift to the bottom all external circumstances which may contribute to prove the negative. Cases of a mixed kind are also sometimes met with; as when a woman will at first resist the advances of a suitor, and even continue her resistance for a time, but afterwards, from the excitement of passion or some other cause, yields to his desire. This is a case, if possible, more puzzling than the former, because marks of violence on the limbs of the female, from her previous struggling, may be evident, which would naturally lead to the supposition that the act had been accomplished by force. We confess that we should be inclined to deal harshly with a man under such circumstances, from the difficulty of understanding what constitutes consent. The act is committed in secret; there are no witnesses; the woman is bruised on the limbs and body; and her person is violated: it is not likely that a formal question of 'Will you consent?' has been put, followed by an answer of yea or nay; and yet, after the employment of so much force, the man defends himself by saying the woman consented, which she denies. The jury alone can determine which is to be credited; but, as we have already said, appearances are strongly in favour of the woman, and a struggle of such violence and duration, followed by coition, amounts, in our opinion, if not to a legal, at least to a moral rape." (P. 583.)

It is not impossible, forsooth! Why, the thing happens every day—in all the cases in which juries acquit, in ninety-nine out of a hundred in which they condemn, and in myriads of cases that never come before a jury at all. And then, after all this stuff, addressed, we should think, not to physiologists, or men of the world, or any one indeed, but petty, very petty jurymen, we have the opinions of Farr, and the Medical Faculty of Leipsic, point-blank the other way, stating the plain truth, namely, that rape is very seldom indeed a possible crime; on which Dr. Beatty gravely says, "It is necessary therefore to be extremely cautious in admitting the truth of accusations, unless the bodily power of the man far exceeds that of the complainant. At the same time, however, we should not entirely agree with the positive opinions just quoted; for we think it possible that, by long-continued violence, intimidation, or other circumstances, the man may ultimately prevail." (P. 590.) So that what was very possible and usual at the beginning of the article becomes almost impossible after half a dozen pages!

It is pleasant to turn from these murderous absurdities to an article by FODÉRÉ on the same subject. He says, "Il est à craindre que la législation ancienne mal interprétée et trop peu précisé, n'ait conduit à l'échafaud beaucoup de victimes innocentes, et qu'en admettant légèrement de semblables accusations comme cela eut lieu jusque vers le milieu du siècle dernier, il n'y ait toujours eu des femmes et des filles assez perverses pour oser dire dans leur courroux contre un ingrat, qu'on les evait prises de force lorsqu'elles s'étaient rendues volontairement. Il y a apparence que ces vengeances par trop cruelles, furent particulièrement communes dans l'Italie méridionale, car nous devons à la législation napolitaine d'avoir la première donné l'éveil sur un abus aussi révoltant, et d'avoir défendu a tous juges de recevoir aucune plainte de viol, à moins qu'il ne fût évident et réal. Jl s'établit dès lors comme une règle même dans les tribunaux français, que l'accusation de ce crime ne devait être admise qu'autant qu'elle était appuyée des quatre faits suivans: 1°. qu'il y avait une inégalité évidente de forces, entre la personne violée et celle de l'accusé; 2°. qu'à presque égalité de forces, il y avait eu une résistance constante et toujours égale de la part de la plaignante; 3°. qu'il était resté sur elle quelques traces de la violence qui lui aurait été faite; 4°, que la crime ayant été commis dans un lieu non solitaire, il était constant qu'elle avait poussée des cris." *(Dict. des Sciences Médicales*, tom. 58, art. *Viol.)*

Dr. Beattie, as we have seen, is too willing to listen to the complaints of those girls who, (to use the words of Fodéré,) in their anger against an ingrate, declare that they were overcome by force, when in fact they surrendered voluntarily; but he gives at length what we may call the evidence for the prisoner in another common class of cases, those in which a false accusation is preferred because a young child has a purulent discharge from the vagina, which is taken for a gonorrhœa. "I can assure you, a multitude of persons have been hanged for such a mistake," says Sir Astley Cooper; and we can assure Dr. Beattie that myriads of persons have been hanged through the mistaken opinions which he cherishes.

Dr. TODD's paper on *Pseudo-morbid Appearances* is useful and interesting; and Dr. GREGORY, physician to the Small-pox Hospital, has given a good account of the disease which he has had such large opportunities of studying.

Many other articles will well repay a diligent perusal; but, far from being able to give a detailed analysis of them, we must content ourselves with mentioning that there is a paper on *the Pulse* by Dr. BOSTOCK, and on *Puerperal Diseases*, by Dr. M. HALL.

